# *Drosophila* Tel2 Is Expressed as a Translational Fusion with EpsinR and Is a Regulator of Wingless Signaling

**DOI:** 10.1371/journal.pone.0046357

**Published:** 2012-09-28

**Authors:** Ji-Hoon Lee, Janice A. Fischer

**Affiliations:** Section of Molecular Cell and Developmental Biology, Institute for Cell and Molecular Biology, The University of Texas at Austin, Austin, Texas, United States of America; University of Dayton, United States of America

## Abstract

Tel2, a protein conserved from yeast to vertebrates, is an essential regulator of diverse cellular processes including telomere maintenance, DNA damage checkpoints, DNA repair, biological clocks, and cell signaling. The *Drosophila* Tel2 protein is produced as a translational fusion with EpsinR, a Clathrin adapter that facilitates vesicle trafficking between the Golgi and endosomes. EpsinR and Tel2 are encoded by a *Drosophila* gene called *lqfR*. *lqfR* is required for viability, and its specific roles include cell growth, proliferation, and planar cell polarity. We find that all of these functions of *lqfR* are attributed entirely to Tel2, not EpsinR. In addition, we find that *Drosophila* LqfR/Tel2 is a component of one or more protein complexes that contain E-cadherin and Armadillo. Moreover, Tel2 modulates E-cadherin and Armadillo cellular dynamics. We propose that at least one of the functions of *Drosophila* Tel2 is regulation of Wingless signaling.

## Introduction

Tel2 is a protein shown to be essential in yeast, nematodes, and vertebrates, that functions in diverse pathways for reacting to a variety of cellular stresses and cues including DNA damage, abnormal mRNAs, nutrient availability, mitogens, and cell cycle progression [1]. Tel2 functions as a co-chaperone with Hsp90 in PIKK complex assembly [2]–[4]. The role of Tel2 in PIKK assembly has been proposed to explain all of its functions, but this point is highly controversial [5]–[7].

The *tel2* gene was identified originally as an essential gene in budding yeast *S. cerevisiae* in a screen for mutants with short telomeres [8]. Genes homologous to *tel2* were found to be essential also in *S. pombe*, *C. elegans*, and mice, but the phenotypes of the mutants and subsequent biochemical studies indicated that Tel2 function is not limited to telomere dynamics [2], [6], [7], [9]–[17].

In the course of a study of the *Drosophila* gene encoding Golgi Epsin or Epsin-Related (EpsinR), we and others [18] discovered that one isoform of *Drosophila* EpsinR is a translational fusion with the only Tel2 coding sequences in *Drosophila*. EpsinR is multi-modular protein conserved from yeast to vertebrates that promotes Clathrin-coated vesicle formation at the trans-Golgi network and endosomes and thereby modulates Golgi-endosome trafficking [19]–[26]. A similar protein conserved in yeast through vertebrates, endocytic Epsin, promotes Clathrin-coated vesicle formation at the plasma membrane [27], [28]. Endocytic Epsin is an essential component of the Notch signaling pathway [29], [30]. As endocytosis and endosomal trafficking play key roles in a variety of signaling mechanisms [31], we were curious whether like endocytic Epsin, Golgi Epsin might be crucial to a particular signaling pathway. To this end, we generated *Drosophila* with loss-of-function mutations in the single EpsinR gene, called *liquid facets-Related* (*lqfR*) [32]. The *lqfR* mutant phenotype is complex; there are defects in planar cell polarity and cell size, proliferation, and patterning [32]. Here we show that these morphological defects of *lqfR* mutants are due entirely to the loss of Tel2 activity. Moreover, we show that the essential Tel2 function in *Drosophila* is at least in part direct regulation of the Wingless signaling pathway.

## Results and Discussion

### Exon 6 of *lqfRa* encodes the *Drosophila* Tel2 homolog

**Figure 1:**
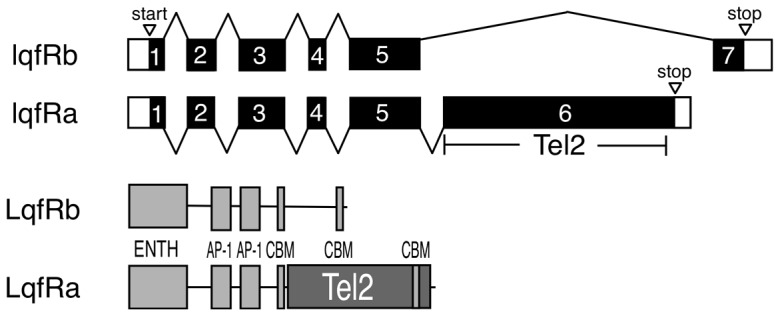
*lqfR* gene products. At top is a diagram of the two *lqfR* mRNAs formed by alternative pre-mRNA splicing. Exons 1–7 are indicated by bars and introns by bent lines. The black region in each transcript is the open reading frame. The larger transcript, *lqfRa*, contains exon6 which encodes Tel2. At bottom are the protein products of each mRNA. ENTH = Epsin N-terminal homology domain, AP-1 = binding motif for the Clathrin adapter AP-1, CBM = Clathrin binding motif.

The *lqfR* gene pre-mRNA is alternatively spliced to generate mRNAs with different C-terminal exons and thus two different proteins, LqfRa (1415 aa) and LqfRb (649 aa) (Fig. 1) [18], [32]. Both LqfRa and LqfRb have structural elements characteristic of Golgi Epsin: the ENTH domain and binding motifs for AP-1 and Clathrin. The larger protein also contains a domain encoded by its LqfRa-specific C-terminal exon 6 (921 aa) that is homologous to Tel2. Tel2 is a Y-shaped protein in the HEAT repeat family of superhelical proteins, in which 32 interacting α-helices are packed to generate two α-solenoids that form the long (21 α-helices) and short (11 α-helices) lines of the Y. Human Tel2 and LqfRa exon6 are 19% identical and 13% similar in amino acid sequence throughout the length of their polypeptide chains (Fig. S1).

The evidence that exon 6 is not a separate gene - that the *lqfRa* splice form truly exists - is convincing. First, there is compelling evidence that exons 5 and 6 are joined in an mRNA; an RT-PCR amplification product containing exon 5 spliced to exon 6 has been generated [32]. Moreover, an exon 3–5 probe hybridizes not only to a species the size of *lqfRb*, but also to a larger mRNA corresponding in size to *lqfRb* that also hybridizes to an exon 6 probe. Second, there is strong evidence that LqfRa and LqfRb proteins both exist; antibodies generated to parts of LqfR that exclude the region encoded by exon 6 hybridize to two bands on Western blots, one corresponding in size roughly to LqfRa, and the other to LqfRb [18], [32].

We were curious to determine whether or not the fusion of the genes for Golgi Epsin and Tel2 was specific to *Drosophila*. We performed two BLAST searches (at www.uniprot.org), one using as a query the amino acid sequence of LqfR exons 1–5, and the other using exon 6. In some species that had clear homologs of both genes, both queries identified the same gene or adjacent genes, indicating that *lqfR* and *tel2* are likely fused in that species. In other species each query identified distinct, non-adjacent genes. Although our analysis was not exhaustive, we did find that the *lqfR* and *tel2* genes were likely fused in all queried *Drosophila* species and also in other insects in the database, but not in yeast, nematodes, nor any vertebrates (data not shown).

### Exon 6 of *lqfRa* is necessary and sufficient for all *lqfR/Tel2* gene functions tested

We found previously [32] that either full-length LqfRa fused at its C-terminus to GFP (LqfRa^FL^-GFP) or a version of the fusion protein that lacks the ENTH domain (LqfRa^ΔENTH^ -GFP), when expressed using *Gal4/UAS* and the ubiquitous *Actin5C-gal4* driver, is sufficient to rescue all of the obvious defects due to loss of *lqfR+* gene activity: these include larval lethality and the absence of imaginal discs. The dispensability of the ENTH domain was not entirely surprising, as endocytic Epsin also functions well without its ENTH domain [33], [34]. However, further structure/function experiments did yield results that were completely unexpected.

**Figure 2:**
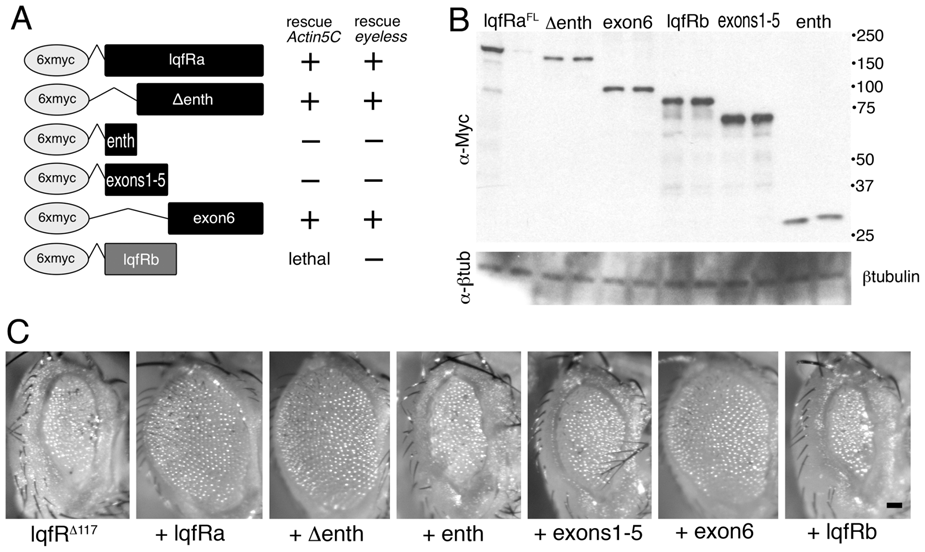
Rescue of *lqfR* null mutant phenotype by *lqfRa* exon 6. (A) At left, the table shows six epitope-tagged proteins expressed in *Drosophila* by a *UAS* transgene. The columns at right show the results when each transgene was expressed in a *lqfR^Δ117^* or *lqfR^Δ117^/Df(3R)Exel6191* background with either an *Actin5C-gal4* or an *eyeless-gal4* driver. +: lethality and externally obvious morphological defects were rescued, − : no rescue. (B) A blot of electrophoresed adult fly protein extracts probed first with antibodies to the Myc tag (α-Myc) and reprobed with antibodies to β-tubulin (α-βtub) as a loading control. The flies contain the *UAS* construct indicated and an *eyeless-gal4* driver. The genotypes of the flies used were: *EGUF/UAS*; *FRT82B lqfR^Δ117^/TM6B*. For each *UAS* construct, two different P element transformant lines were tested. Note that one of the *UAS-lqfRa^FL^* lines expressed little or no protein and this line also failed to rescue the *lqfR^Δ117^* mutant phenotype. The numbers at the right of the blot indicate the positions of corresponding size markers (kD). (C) Light microscope images of the eyes of adult flies. The flies are *lqfR^Δ117^/lqfR^+^* and their eyes are *lqfR^Δ117^* homozygous clones. The fly at the very left has no *UAS* transgene and the others contain a copy of the *UAS* transgene indicated, expressed by *eyeless-gal4*. The genotypes of the flies were: *EGUF/UAS*; *FRT82B lqfR^Δ117^/FRT 82B GMR-hid*. scale bar: ∼50 µm.

First, we generated five *UAS* transgenes in P element vectors, in which full-length (FL) *lqfRa* or four deletion derivatives were tagged with 6xmyc epitope coding sequences at their 5′ ends (Fig. 2A) and used them to transform *Drosophila*. Each transgene was tested for its ability when expressed with *Actin5C-gal4* to substitute for the endogenous *lqfR* gene. The results obtained by expressing LqfRa^FL^-GFP or LqfRa^ΔENTH^-GFP described above were recapitulated by 6xmyc-LqfRa^FL^ and 6xmyc-LqfRa^ΔENTH^: expression of either protein rescued *lqfR* null mutants to wild-type (Fig. 2A). In contrast, neither the ENTH domain alone (6xmyc-LqfR^ENTH^) nor exons 1–5 alone (6xmyc-LqfR^ex1-5^) had any rescuing activity (Fig. 2A). This was not due to a failure of transgene expression as the 6xmyc-LqfR^ENTH^ and 6xmyc-LqfR^ex1-5^ proteins accumulated in the flies to levels at least as high as 6xmyc-LqfRa^ΔENTH^ (Fig. 2B). The most remarkable result was that exon 6 alone (6xmyc-LqfR^exon6^) rescued *lqfR* null mutants to wild-type (Fig. 2A). In summary, we found that expression of exon 6, which contains only the Tel2-like region of LqfRa, was sufficient to rescue the imaginal disc proliferation and patterning defects of *lqfR* null mutants and no other portions of LqfRa were able to provide any rescuing activity independently.

The results so far predict that LqfRb, which does not contain exon 6, would not have any rescuing activity. We could not test LqfRb in the assay described above because unexpectedly, expression of *UAS-6xmyc-lqfRb* with *Actin5C-gal4* was lethal. To overcome this obstacle, we expressed *6xmyc-lqfRb* in the eye only, and asked if the eye morphology defects in eyes with no LqfR protein in otherwise normal flies (*lqfR^Δ117^/lqfR^+^* flies with homozygous *lqfR^Δ117^* eyes generated using *FRT/GMR-hid*) were rescued. *lqfR^Δ117^* eyes are tiny and rough compared to wild-type (Fig. 2C). Flies expressing 6xmyc-LqfRb with *ey-gal4* were viable, and we found that 6xmyc-LqfRb had no rescue activity in the eye (Fig. 2A,C). As controls, we tested the four transgenes described above and we found the same results with those as we did with the *Act5C-gal4* driver: 6xmyc-LqfRa^FL^, 6xmyc-LqfRa^ΔENTH^, or 6xmyc-LqfR^exon6^ rescued *lqfR^Δ117^* eyes to wild-type and 6xmyc-LqfR^ex1-5^ did not rescue (Fig. 2A,C). The failure of 6xmyc-LqfRb to rescue was not due to failure of protein expression, as LqfRb protein accumulated to levels similar to those of LqfR^exon6^ (Fig. 2B). The inability of *lqfRb* to complement the *lqfR* mutant phenotype is consistent with the finding that exon 6 alone of *lqfRa* is sufficient to do so.

We conclude that Golgi Epsin and Tel2, although fused in LqfRa, are independent protein functions. Moreover, the external morphology and lethality aspects of the mutant phenotype described for *lqfR* null mutants reflects only the loss of Tel2 activity, and not the loss of Golgi Epsin. We therefore propose renaming the *lqfR* gene *lqfR/tel2*.

### The Tel2-like portion of LqfRa encoded by exon 6 expressed alone is mainly nuclear

**Figure 3:**
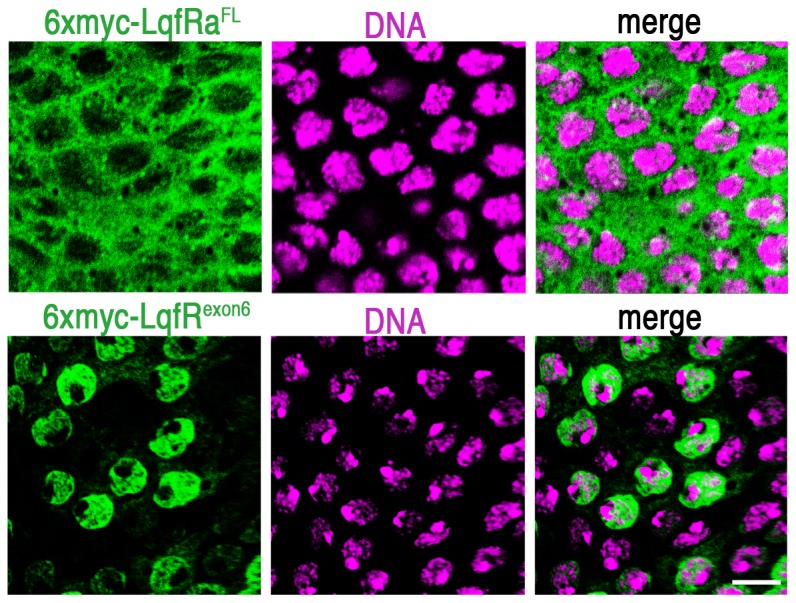
Subcellular localization of Myc-tagged LqfR proteins. Confocal microscope images of third instar larval eye disc tissue from two different discs (each row is a single disc) are shown. The portion of the eye disc shown is the peripodial epithelium, a layer of cells that lies atop the cell layer that forms the retina. The peripodial cells are large and flat the nuclei and cytoplasm are distinguished more easily than in the retinal cells. The discs were immunostained with antibodies to the Myc epitope (green) and the DNA stain TOPRO3 (purple). The Myc-tagged proteins indicated were expressed by *UAS* transgenes using an *Actin5C-gal4* driver. scale bar: ∼10 µm.

Using either of two different polyclonal antibodies, one to LqfR exons 1–5 and the other to an ENTH-less LqfRb, LqfR was shown to colocalize with Golgi markers in the eye and elsewhere [18], [32]. We were curious to know where the truncated protein consisting of LqfRa exon 6 alone (6xmyc-LqfR^exon6^) accumulates in the cell. Full length 6xmyc-LqfRa^FL^ monitored with anti-Myc had a cytoplasmic localization pattern similar to that of endogenous LqfR and other Golgi markers (Fig. 3) [18], [32]. By contrast, 6xmyc-LqfR^exon6^ was mainly nuclear (Fig. 3). Co-labeling with TOPRO3 suggests that exon6 is at the nuclear envelope because it does not colocalize with DNA, but surrounds it (Fig. 3). Further experiments are required to determine whether Tel2 is localized to the nuclear side or the cytoplasmic side of the nuclear envelope. Nevertheless, the majority of the Tel2-like portion of LqfRa does not localize to the Golgi as the full length LqfR protein does, and yet it is sufficient to rescue the *lqfR/Tel2* mutant phenotype. The implication is that the essential *lqfRa/tel2* gene function may not be at the Golgi.

These results raise a question: as LqfRa/Tel2 contains the amino acids encoded by exons 1–5 and exon 6, why does the antibody to LqfR exons 1–5 not include the labeling pattern of 6xmyc-LqfR^exon6^ – that is the nuclear envelope? One explanation may lie in the observation that LqfRb, which lacks the exon 6-encoded amino acids, is the majority of the LqfR protein present in eye discs [32]. Thus, the antibody to exons 1–5 may be detecting LqfRb only. Alternatively, it is possible that the exon 6-encoded Tel2 region of LqfRa is cleaved post-translationally from the N-terminus of the protein, so that the antibody to exons 1–5 does not detect exon 6-encoded protein. Yet another possibility is that is LqfRa/Tel2 normally shuttles between the cytoplasm and the nucleus and the 6xmyc-Tel2 protein fusion is retained at the nuclear envelope abnormally. The generation of an antibody specific to the Tel2-like region of LqfRa might help to distinguish among these alternatives.

### Wingless pathway genes interact strongly with *lqfR/tel2*

The specific cell growth and patterning defects in *lqfR/Tel2* mutants are suggestive of defects in a variety of different signaling pathways [32]. Wingless signaling, for example, regulates both cell proliferation and patterning in the eye [35]. Wingless regulates initiation of the wave front of eye morphogenesis called the morphogenetic furrow. In addition, Wingless expressed at the lateral margins of the eye disc forms a gradient that results in formation of a dorsal/ventral midline called the equator about which the facets, or ommatidia, are mirror-image symmetrical. Separation of eye and head cuticle tissue also requires Wingless. As the *lqfR/tel2* mutant phenotype includes defects in morphogenetic furrow movement and planar cell polarity in both the eye and wing [32], it seemed reasonable that the function of *lqfR/tel2* could somehow relate to the Wingless pathway.

**Figure 4:**
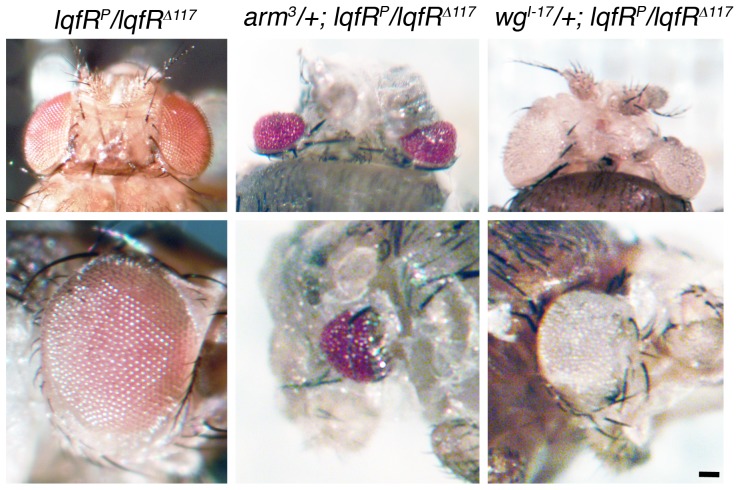
Genetic interactions between *lqfR*, *armadillo*, and *wingless*. Shown are light microscope images of adult fly heads (dorsal view, top row), and eyes (bottom row). The genotypes of each column are indicated at top. The same fly is shown in the top and bottom rows. scale bar: ∼50 µm.

We tested two genes encoding core components of the Wingless pathway, *wingless* and *armadillo*, for interactions with *lqfR/Tel2*. Wingless ligand binds its receptor Frizzled which results in accumulation in the nucleus of the transcriptional regulator Armadillo [36]. We found strong genetic interactions between *lqfR/tel2* and each of the two Wingless pathway genes. Heterozygotes for a hypomorphic allele and a null allele of *lqfR* (*lqfR^P^*/*lqfR^Δ117^*) are semi-viable and the adult escapers may have normal, slightly roughened, or kidney-shaped eyes [32] (Fig. 4). Flies that were *lqfR^P^*/*lqfR^Δ117^* and also heterozygous for a loss-of-function allele of *armadillo* (*arm^3^* or *arm^8^*) died in their pupal cases. (*arm-/arm+* flies appear wild-type.) The pupae had small or absent eyes, small head capsules, and also had morphological defects in the head cuticle, wings, and legs (Fig. 4). *wingless* loss-of-function mutations (*wg^I-17^* or *wg^I-8^*) had similar but weaker effects when heterozygous in a *lqfR^P^*/*lqfR^Δ117^* background; there were viable adult escapers with severely defective eyes varying from kidney-shaped to nearly absent, and also with defects in the head cuticle (Fig. 4). (*wg−/wg+* animals appear wild-type.) These strong genetic interactions suggest that *lqfR/tel2* may function in the Wingless signaling pathway.

### Wingless target gene expression depends to some extent on *lqfR/tel2* function

The dominant enhancement of *lqfR/tel2* mutant phenotypes by loss of function mutations in Wingless pathway genes suggests that *lqfR/tel2* facilitates Wingless pathway activation. To test this idea, we generated *lqfR/tel2* null clones in eye discs and monitored expression of the Wingless target genes *dachsous* (*ds*) [36], [37] and *optomotor blind* (*omb*) [38]. Weak effects on target gene expression were apparent in both cases. As the effects on *ds* expression was stronger, this data is shown below.

**Figure 5:**
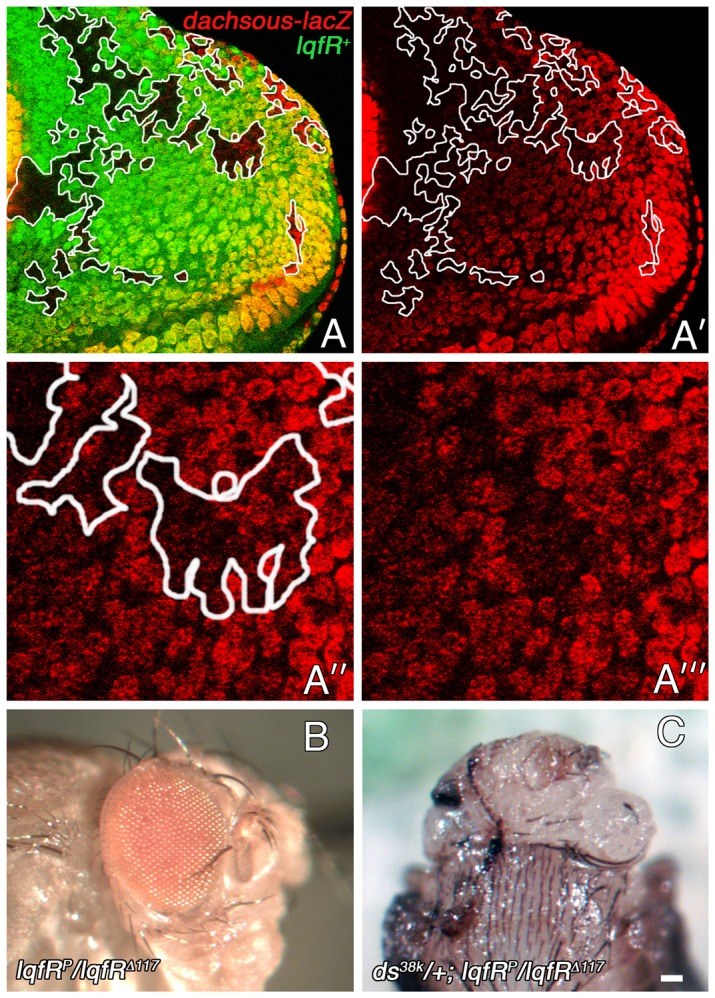
Genetic interactions between *lqfR* and *dachsous*. (A-A′″) Confocal microscope images of an eye disc immunostained with antibodies to β-galactosidase are shown. The disc expresses GFP in all cells except for *lqfR^Δ117^* homozygous clones. The genotype is *ds-lacZ/+*; *FRT82B lqfR^Δ117^/FRT82B ubi-gfp*. (A′) Clones are outlined. (A″,A″′) Enlargements of part of A′ which shows that *ds-lacZ* expression levels are lower in *lqfR^Δ117^* clones than in adjacent wild-type tissue. (B) A light microscope image of an eye from an adult fly hypomorphic for *lqfR* is shown. (C) The head (dorsal view) of a pupa that will not eclose dissected from its pupal case. *ds^38k^/ds^+^* animals (not shown) appear wild-type. scale bar: ∼10 µm in A,A′; ∼5 µm in A″, A″′; ∼100 µm in B,C.

The *dachsous* gene encodes an atypical cadherin adhesion protein involved in cell polarity and cell growth and is a transcriptional target of Arm [37], [38]. *ds-lacZ* enhancer trap lines express β-galactosidase in response to Wg pathway activation [38]. Wingless ligand is expressed in the dorsal- and ventral-most margins of the eye disc and the protein forms a gradient with its lowest point at the dorsal/ventral axis (the equator) [40]. β-galactosidase expression by *ds-lacZ* reflects the Wg gradient [39]. We found that *ds-lacZ* expression was reduced in *lqfR/tel2* null clones (Fig. 5). Moreover, we found that a *dachsous* loss-of-function mutation, *ds^38K^*, is as strong a dominant enhancer of the *lqfR^P^*/*lqfR^Δ117^* mutant phenotype as are *armadillo* mutations (Fig. 5). These results suggest that in the absence of LqfR/Tel2, Wg signaling is less efficient than it is normally.

The effects of *lqfR/tel2* loss of function on Wingless target gene expression are weaker than expected based on the dramatic genetic interactions between *lqfR/tel2* mutations and mutations in *arm*, *wg*, or *ds*. (We also monitored expression of the Wingless target gene *senseless* (*sens*) [41] in wing disc *lqfR/tel2* null clones but could see no effect). One possible explanation is that the effects of a modest decrease in Wingless signaling are amplified by downstream effects on other signaling pathways. Therefore, the combined effect of losses in several signaling pathways in *lqfR/tel2* mutants could account for the striking genetic interactions.

### Plasma membrane levels of E-cadherin and Armadillo increase in the absence of *lqfR/Tel2* activity

In a mutagenesis screen for dominant enhancers of the *lqfR/tel2* mutant eye phenotype (Lee et al., manuscript preparation), we identified loss-of-function alleles of *polychaetoid*, which encodes the *Drosophila* homolog of vertebrate ZO-1 [42]. in ZO-1/Polychaetoid is present at tight junctions and adherens junctions, where it connects other proteins present there to the actin cytoskeleton [42]–[44]. Although the mechanism is unclear, loss of Polychaetoid in *Drosophila* results in increased accumulation of the cell adhesion protein E-cadherin at the plasma membrane [43].

The transmembrane protein E-cadherin is a central component of adherens junctions through homotypic interactions between E-cadherin extracellular domains on adjacent cells [45]. The intracellular domain of E-cadherin binds proteins, including Armadillo and α-catenin, which are essential for E-cadherin's function as a cell adhesion protein [46], [47] Because E-cadherin binds Armadillo, E-cadherin function effects Wingless signaling [48]. Notably, E-cadherin overexpression antagonizes Wingless signaling, presumably by preventing Armadillo from entering the nucleus [49], [50].

**Figure 6:**
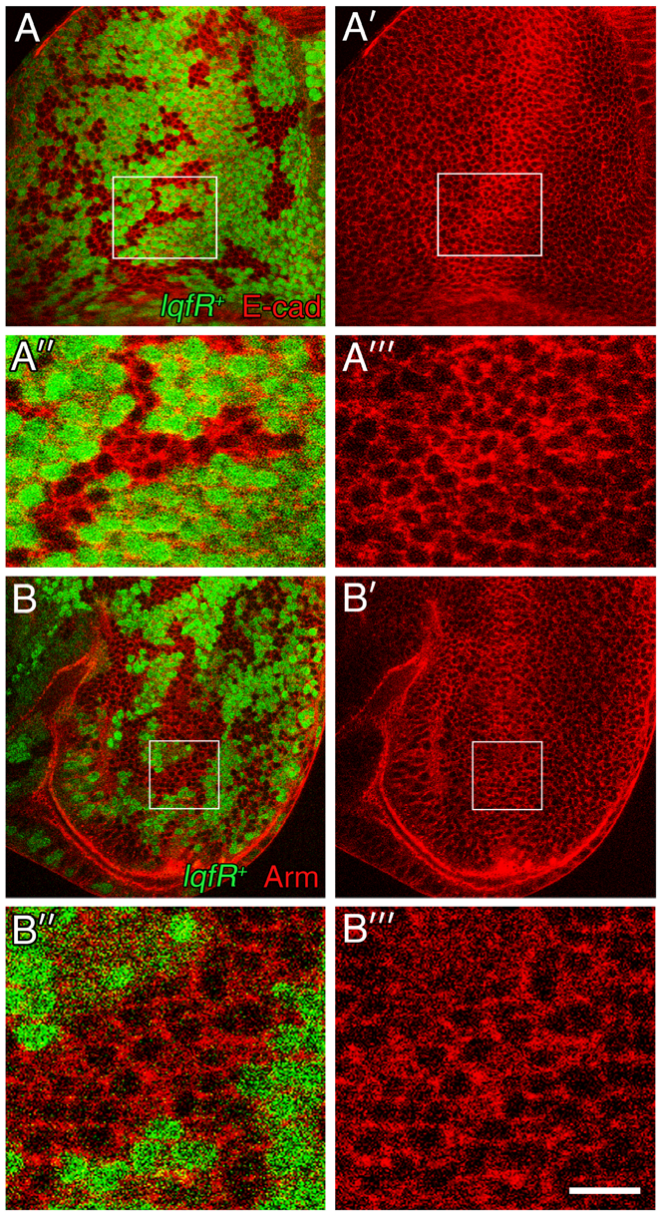
E-cadherin and Armadillo protein accumulation in *lqfR*- clones. (A,A′) Confocal microscope images of an eye disc immunostained with E-cadherin antibodies (red). *lqfR*- clones are marked by the absence of GFP (green). (A″,A″′) Enlargements of the boxed regions in A and A′. (B,B′) Confocal microscope images of an eye disc immunostained with Armadillo antibodies (red). *lqfR*- clones are marked by the absence of GFP (green). The genotype for both experiments is *ey-flp; FRT82B lqfR^Δ117^/FRT82B ubi-gfp*. scale bar: ∼40 µm in A,A′,B,B′; ∼10 µm in A″, A″′,B″,B″′.

We wondered whether the genetic interaction between *polychaetoid* and *lqfR/tel2*, which suggests that both genes facilitate Wingless signaling, could be explained by the effect of Polychaetoid on E-cadherin levels. To test this hypothesis, first we asked whether E-cadherin levels, which increase in the absence of Polychaetoid, were also elevated in eye disc clones lacking LqfR/Tel2. We found that E-cadherin levels do indeed increase in *lqfR/tel2* null clones; this effect appears most dramatic near the morphogenetic furrow where the highest levels of E-cadherin accumulate normally (Fig. 6). In discs where either 6xmyc-LqfRa^FL^ or 6xmyc-LqfR^exon6^ were overexpressed, *lqfR/tel2* null clones had the same levels of E-cadherin as surrounding wild-type tissue, confirming that the effect on Cadherin is mediated by Tel2 (Fig. S2). Like E-cadherin levels, Armadillo levels at the plasma membrane also are higher than usual in the absence of LqfR/Tel2 (Fig. 6). We conclude that the Tel2-like portion of LqfRa modulates Wingless signaling through an effect, either direct or indirect, on E-Cadherin levels and Armadillo localization.

### LqfR/Tel2 interacts physically with E-cadherin, Armadillo and α-catenin

**Figure 7:**
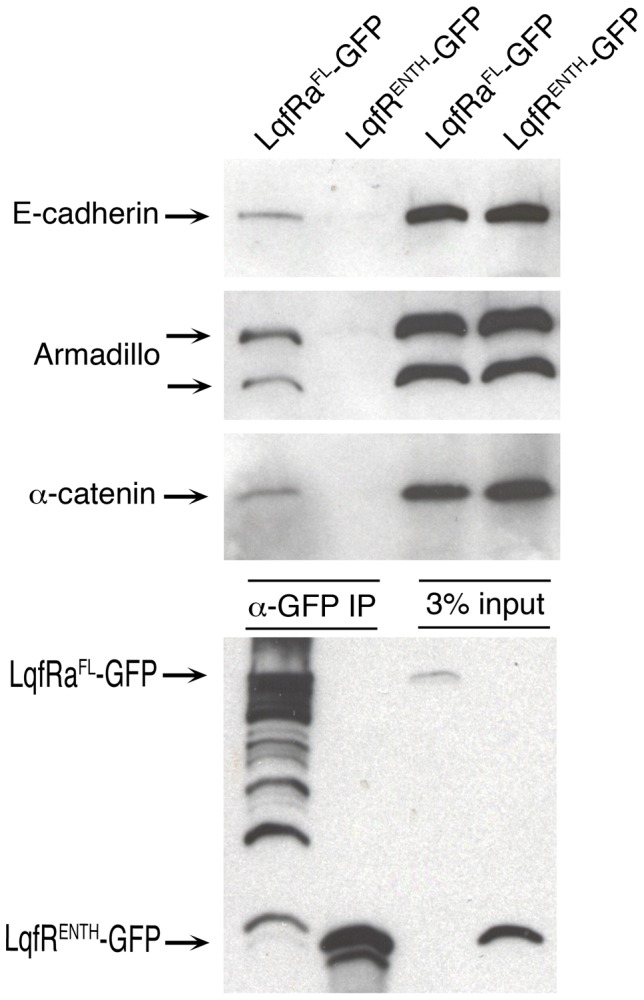
Coimmunoprecipitation of LqfRa and Wingless pathway proteins. Shown is a blot of protein extracts, before and after immunoprecipitation, from embryos expressing either LqfRa^FL^-GFP or LqfRa^ENTH^-GFP as a negative control. The LqfR protein fusions were expressed from *UAS* transgenes using an *Actin5C-gal4* driver. The two leftmost lanes (α-GFP IP) are immunoprecipitates using GFP antibodies, and the rightmost lanes (3% input) are aliquots of the protein extracts used, loaded to show that equivalent amounts of protein were present in each extract subjected to immunoprecipitation.

To determine whether or not the effect of LqfR/Tel2 on E-cadherin and Armadillo is direct, we asked whether LqfR/Tel2 is present in a complex with either protein. We also tested for physical interactions between LqfR/Tel2 and the adherens junction protein α-catenin, which binds Armadillo. First, we used antibodies to GFP to immunoprecipitate LqfRa-GFP from fly embryos that overexpress it (*Actin5C>lqfRa-gfp*). Next, using antibodies to each of the three proteins on blots, we determined whether E-cadherin, Armadillo, or α-catenin were also present in the precipitate. We found that each of the three proteins co-immunoprecipitated with LqfRa-GFP (Fig. 7). The adherens junction proteins are not binding to GFP because we did the same experiment with embryos that overexpress the ENTH domain only fused to GFP (LqfR^ENTH^-GFP) and we found that LqfR^ENTH^-GFP did not coimmunoprecipate with any of the three proteins (Fig. 7). Moreover, the correlation between rescue of the *lqfR/tel2* mutant phenotype and binding to the adherens junction proteins (LqfRa-GFP both rescues and binds and LqfR^ENTH^-GFP does neither) suggests that the interaction between LqfR/Tel2 and E-cadherin, Armadillo and α-catenin may be relevant to the *lqfR/tel2* mutant phenotype and that the effect of LqfR/Tel2 on adherens junctions is direct. Further experiments are required to determine whether or not all four proteins are present in a single complex.

### LqfR/Tel2 is not required for Wntless-mediated Wingless secretion

**Figure 8:**
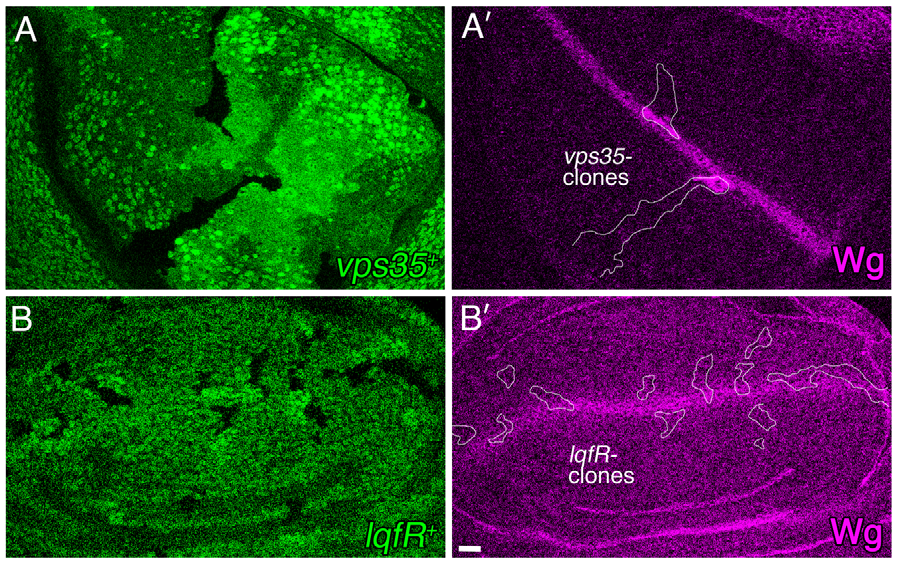
Wg protein secretion in *lqfR*- clones. Shown are confocal microscope images of two third instar larval wing discs immunostained with Wg antibodies (purple). Wingless is expressed and secreted by a stripe of cells at the dorsal/ventral boundary. Homozygous mutant clones are marked by the absence of GFP (green). (A,A′) A wing disc with *vps35^E42^* homozygous clones, outlined in white in A′. The genotype is *hs-flp; FRT42D vps35^E42^/FRT42D ubi-gfp*. (B,B′) A wing disc with *lqfR^Δ117^* mutant clones, outlined in white in B′. The genotype is *hs-flp; FRT82B lqfR^Δ117^/FRT82B ubi-gfp*. scale bar: ∼10 µm.

Wingless secretion requires the transmembrane protein Wntless, which binds to Wingless at the Golgi and guides it to the plasma membrane. After releasing Wingless, Wntless is endocytosed and trafficked back to the Golgi. Retrograde trafficking of Wntless from endosomes to Golgi is essential for Wingless secretion and requires the retromer complex. Cell clones lacking retromer complex proteins cannot secrete Wingless and instead Wingless accumulates inside the cells [51]–[53]. In human cultured cells, EpsinR is required for retromer complex function [54], [55]. We have shown that loss of Tel2 activity, and not loss of Golgi Epsin, is the reason why Wingless signaling falters in *lqfR/tel2* mutants. Nevertheless, as Golgi Epsin is expected to be required for Wingless secretion, we tested whether or not *lqfR/tel2* activity is required. Port et al., 2008 showed that cell clones in the wing disc lacking the retromer protein Dvps35 accumulate Wingless and we were able to replicate this result (Fig. 8A,A′). In contrast, *lqfR/tel2* null clones have wild-type Wingless levels (Fig. 8B,B′) and we infer that Wingless is secreted normally from the mutant cells. This result is consistent with the observation that Tel2 expression rescues the *lqfR/tel2* null mutant phenotype. Moreover, we conclude that in *Drosophila*, Golgi Epsin is not always required for retromer complex function.

## Conclusions

Many of the results presented here were unexpected and raise questions that remain to be answered. First, we were surprised to find that in *Drosophila*, EpsinR and Tel2 proteins are fused. The gene fusion appears to have no consequence for the essential function of the locus in *Drosophila*. Whether or not the two parts of the protein function together in other contexts, for example during oogenesis or in redundant functions undetectable in our experiments, is unknown. It was also unexpected that only the Tel2 function is essential in *Drosophila*; EpsinR is not essential for viability in flies and EpsinR is not required for the function of the retromer complex in Wntless recycling. Whether retrograde trafficking of Wntless is the exception, or whether retromer complex function is generally EpsinR-independent in *Drosophila* remains to be determined.

**Figure 9:**
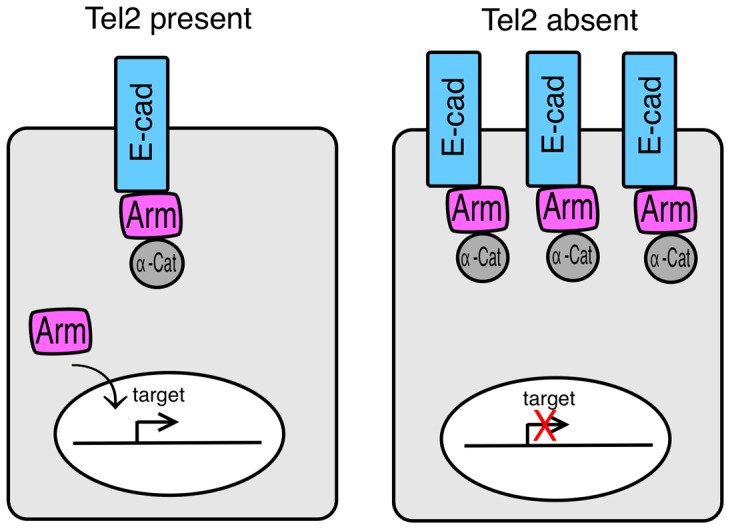
The effect of Tel2 on Wingless signaling. A model for how Wingless signaling is compromised in the absence of Tel2 is illustrated. We speculate that in the absence of Tel2, increased E-cadherin at the plasma membrane sequesters Armadillo (Arm) so that little remains free in the cytoplasm to enter the nucleus in response to Wingless signaling.

At least one aspect of Tel2's essential function in *Drosophila* is to promote Wingless signaling through modulation of adherens junction proteins. Whether or not the *lqfR/tel2* mutant phenotype in its entirety reflects a failure of Wingless signaling is yet unclear. There are mutant phenotypes of *lqfR* mutant flies that are not easily explained by a loss of Wingless signaling [32]. Moreover, recent results link the function of LqfR/Tel2 to a PIKK complex [56]. Nevertheless, the results presented suggest that the association of Tel2 with adherens junction proteins prevents the accumulation of excess E-cadherin at the plasma membrane which would otherwise sequester Armadillo and prevent efficient Wingless signaling (Fig. 9). Further experiments are required to determine precisely how Tel2 activity affects E-cadherin levels. It is interesting that loss of Tel2 activity in *C.elegans* phenocopies, at least in part, loss of Wnt signaling proteins rather than loss of PIKK activities [7]. The results presented here pave the way for genetic experiments to determine whether or not Tel2 activity in *Drosophila* always involves PIKK complexes.

## Materials and Methods

### *Drosophila* strains

The following mutations were used: *lqfR^P^* (FBal0230310), *lqfR^Δ117^* (FBal0191038), *Df(3R)Exel6191* (Fab0038246), *ds^38k^* (FBal0028156), *arm^3^* (FBal0000712), *arm^8^* (FBal0000716), *vps35^E42^* (FBal0221801), *wg^I-17^* (FBal0018509). The following transgenic lines were used: *Actin5C-gal4* (FBti0012293), *ey-gal4* (FBti0012711), *EGUF* (FBti0012712, FBti0012284), *hs-flp* (FBti0015982), *ey-flp* (FBti0015982), *FRT82B* (FBti0002074), *FRT42D* (FBti141188), *ubi-gfp* (FBti0012695, FBti0015577), *ds-lacZ* (FBal0045522), *GMR-hid* (FBti0012710), *UAS_t_-lqfRa^FL^-gfp* [32], *UAS_t_-lqfRa^ENTH^-gfp* [57]. The following transgenic lines (on chromosome 2) were generated: *UAS_t_-6xmyc-lqfRa^FL^, UAS_t_-6xmyc-lqfRa^ΔENTH^, UAS_t_-6xmyc-lqfRa^exons1-5^*, *UAS_t_-6xmyc-lqfRa^exon6^*, *UAS_t_-6xmyc-lqfRb*. Chromosomes used are indicated in the figure legends.

### Transgene construction and transformation

DNA fragments for four of the *lqfR* P element constructs were generated as follows. First, the template *pUAS_t_-lqfRa-gfp* [32] was used with the following primer pairs to amplify four different products:

*lqfRa^FL^*:

F: 5′- CACCGTGGATAAATTCATCAGCATGTGGAAAG

R: 5′- TTAGGCAGCCTGTTCCATGGCG

*lqfRa^ΔENTH^*:

F: 5′- CACCGTGGATAAATTCATCAGCATGTGGAAAG

R: 5′-TTAGGCAGCCTGTTCCATGGCG

*lqfRa^exon6^*:

F: 5′-CACCGCTGTTGAAGAGCAGTTGGCATCC

R: 5′-TTAGGCAGCCTGTTCCATGGCG

*lqfRb*:

F: 5′- CACCATGCACGTGGTGGATAAATTCATCAG

R: 5′- TTATCATTGAAACAAGTCGAATGCCG

The PCR products were ligated into *pENTR/D-TOPO* (Invitrogen). A DNA fragment containing *lqfRa^exons1-5^* was generated by modifiying *pENTR-lqfRa^FL^*. The plasmid was restricted with *Stu I* and *Sna BI* and then religated, thus removing the final 38 bp of exon 5 and most of exon 6, except for 402 bp at the 3′ end. The *lqfR* fragments in *pENTR-lqfR* were transferred to the *pTMW* vector (Drosophila Genomics Resource Center, #1107) by using site-specific recombination (http://emb.carnegiescience.edu/labs/murphy/Gateway%20vectors.html). The sequence of each *pTMW-lqfR* plasmid was verified. P element-mediated transformation of *yw* was performed by Genetivision (Houston, TX).

### Analysis of eyes

For immunofluorescence, eye discs were fixed in PEMS and antibody incubations and washes were in PBST as described [58]. Primary antibodies used (DSHB = Developmental Studies Hybridoma Bank): rat anti-E-cadherin (DSHB∶DCAD2, used 1∶100), mouse anti-Armadillo (DSHB∶N27A1, used 1∶100), mouse anti-β-balactosidase (DSHB∶40-1a, used 1∶50), mouse anti-Myc (Santa Cruz Biotechnology∶sc-40, used 1∶20), mouse anti-Wingless (DSHB∶4D4, used 1∶100). Secondary antibodies were as in Lee et al., 2009. Confocal microscopy of eye discs, light microscopy of adult eyes, and image processing was as described [32].

### Protein blot in Figure 2

Protein extracts of 2 adult flies containing one copy each of the transgene indicated and the *ey-gal4* driver were made by homogenizing and boiling in 2× Laemmli Buffer. After SDS-PAGE, Western blotting and probing was performed as described [59]. Primary antibodies were mouse anti-β-tubulin (DSHB∶E7, 1∶100), and anti-Myc (Santa Cruz Biotechnology∶sc-40, used 1∶500) [32].

### Immunoprecipitation

Protein extracts were prepared from *Act>lqfRa-gfp* and *Act>lqfR^ENTH^*-*gfp* embryos: GFP-positive embryos were homogenized in 100 µl lysis buffer (1% NP40, 0.5% deoxycholate, 1 mM DTT, 150 mM NaCl, 50 mM Tris pH 8.0 with protease inhibitor cocktail [Roche, complete-mini, EDTA-free] and 2 mM PMSF). Lysis buffer (300 µl) was added followed by centrifugation at 12,000 rpm at 4°C. A 300 µl aliquot was removed and mixed with 20 µl of a 50% slurry of GFP-trapA (Chromotek) and a 10 µl aliquot was mixed with 2× SDS loading buffer as a loading control. After incubating 2 hrs. with mild shaking at 4°C, the 300 µl aliquot was spun down, the pellet collected and washed for 5 min. with shaking in 1 ml lysis buffer, and then washed again for 10 min. with shaking in 1 ml of 500 mM NaCl. The pellet was washed 4 times more in 1 ml of 500 mM NaCl and then mixed with 20 µl of 2× Laemmli Buffer. Each sample was boiled for 5 min, microfuged, and the supernatant subjected to SDS-PAGE in a 7.5% gel. Western blotting was performed as described (Chen et al., 2002). Primary antibodies were: rat anti-E-cadherin (DSHB∶DCAD2, used 1∶1000), mouse anti-Armadillo (DSHB∶N27A1, used 1∶500), rat anti-α-catenin (DSHB∶DCAT-1, used 1∶100), rat anti-GFP (Chromotek∶3H9, used 1∶1000). Secondary antibodies were from Santa Cruz Biotechnology and used at 1∶5000: goat anti-rat HRP , goat anti-mouse HRP, goat anti-rat HRP.

## Supporting Information

Figure S1 **Amino acid sequence alignment of human and yeast Tel2 and** ***Drosophila*** **LqfR-exon 6.** The amino acid sequences of *H. sapiens* Tel2, *D. melanogaster* LqfR exon 6, and *S. cerevisiae* Tel2 were aligned using MacVector and the results are shown. *H. sapiens* vs. *S. cerevisiae*: aligned length = 850, gaps = 23, identities = 116 (13%), similarities = 102 (12%). *H. sapiens* vs. *D. melanogaster*: aligned length = 929, gaps = 15, identities = 181 (19%), similarities – 158 (17%). *D. melanogaster* vs. *S. cerevisiae*: aligned length = 924, gaps = 18, identities = 110 (11%), similarities = 121 (13%).
(TIF)

Figure S2 **Rescue of E-cadherin accumulation abnormality in** ***lqfR*** **- clones by transgene expression.** Confocal microscope images of three third instar larval eye discs immunostained with antibodies to E-cadherin (red). *lqfR*- clones are marked by the absence of GFP (green). The images at bottom are identical to the ones at the top except only the red layer is shown and the clone is outlined. (A–C′) The discs express the transgenes indicated. The genotype is *ey-flp; FRT82B lqfR^Δ117^/FRT82B ubi-gfp* in all panels, with the addition of *Act5C-gal4, UAS-lqfRa/ +* (B,B′) and *Act5C-gal4, UAS-lqfRa^exon6^/ +* (C,C′) on chromosome 2. scale bar: ∼10 µm in A–B′; ∼25 µm in C,C′
(TIF)
