# Supplementary figures and images for: Drosophila Tel2 Is Expressed as a Translational Fusion with EpsinR and Is a Regulator of Wingless Signaling
Source: PLoS One. 2012 Sep 28;7(9):e46357. doi: 10.1371/journal.pone.0046357 (PMC3460857; doi:10.1371/journal.pone.0046357)

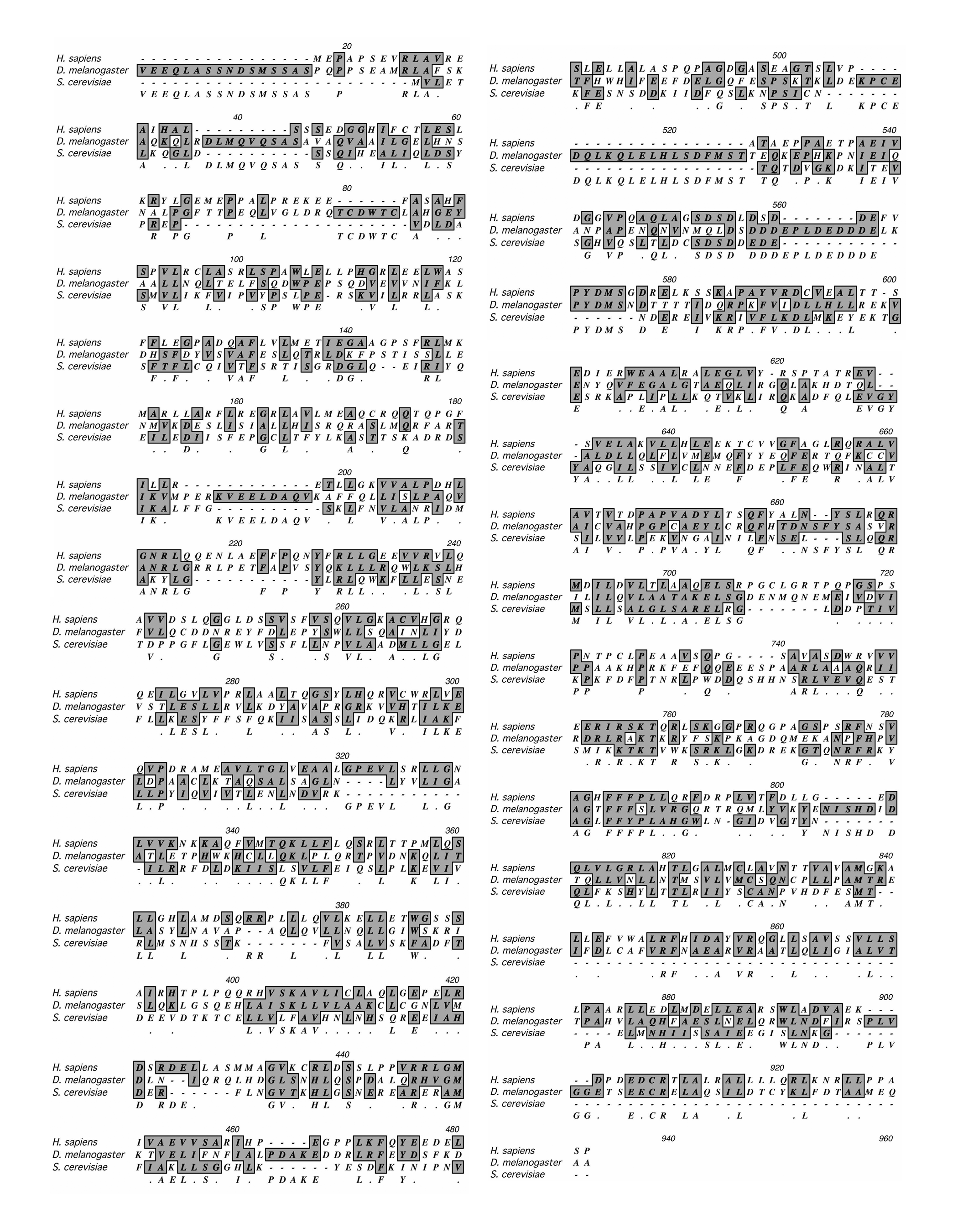

Supplement: Figure S1 — Amino acid sequence alignment of human and yeast Tel2 and Drosophila LqfR-exon 6. The amino acid sequences of H. sapiens Tel2, D. melanogaster LqfR exon 6, and S. cerevisiae Tel2 were aligned using MacVector and the results are shown. H. sapiens vs. S. cerevisiae: aligned length = 850, gaps = 23, identities = 116 (13%), similarities = 102 (12%). H. sapiens vs. D. melanogaster: aligned length = 929, gaps = 15, identities = 181 (19%), similarities – 158 (17%). D. melanogaster vs. S. cerevisiae: aligned length = 924, gaps = 18, identities = 110 (11%), similarities = 121 (13%). (TIF) [file pone.0046357.s001.tif]

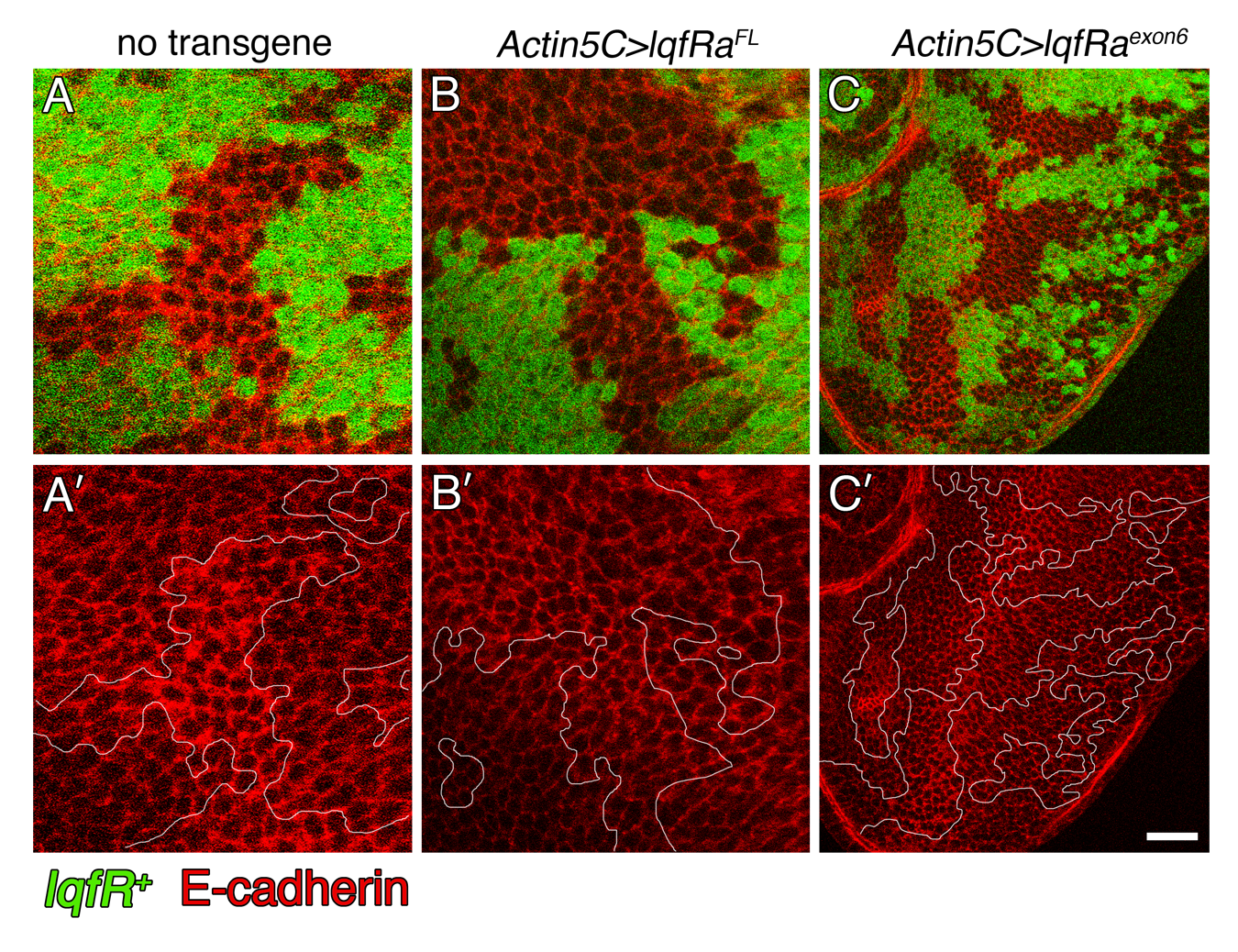

Supplement: Figure S2 — Rescue of E-cadherin accumulation abnormality in lqfR - clones by transgene expression. Confocal microscope images of three third instar larval eye discs immunostained with antibodies to E-cadherin (red). lqfR- clones are marked by the absence of GFP (green). The images at bottom are identical to the ones at the top except only the red layer is shown and the clone is outlined. (A–C′) The discs express the transgenes indicated. The genotype is ey-flp; FRT82B lqfRΔ117/FRT82B ubi-gfp in all panels, with the addition of Act5C-gal4, UAS-lqfRa/ + (B,B′) and Act5C-gal4, UAS-lqfRaexon6/ + (C,C′) on chromosome 2. scale bar: ∼10 µm in A–B′; ∼25 µm in C,C′ (TIF) [file pone.0046357.s002.tif]
